# Invasive behavior of ulcerative colitis-associated carcinoma is related to reduced expression of CD44 extracellular domain: comparison with sporadic colon carcinoma

**DOI:** 10.1186/1746-1596-6-30

**Published:** 2011-04-07

**Authors:** Tetuo Mikami, Tsutomu Yoshida, Yoshiko Numata, Masaomi Kikuchi, Kayo Araki, Norihiro Nakada, Isao Okayasu

**Affiliations:** 1 Department of Pathology, Kitasato University School of Medicine, Sagamihara, Japan

## Abstract

**Background:**

To elucidate relations of invasion of ulcerative colitis (UC)-associated carcinoma with its prognosis, the characteristics of invasive fronts were analyzed in comparison with sporadic colonic carcinomas.

**Methods:**

Prognoses of 15 cases of UC-associated colonic carcinoma were compared with those of sporadic colon carcinoma cases, after which 75 cases of sporadic invasive adenocarcinoma were collected. Tumor budding was examined histologically at invasive fronts using immunohistochemistry (IHC) of pancytokeratin. Expressions of beta-catenin with mutation analysis, CD44 extracellular domain, Zo-1, occludin, matrix matalloproteinase-7, laminin-5γ2, and sialyl Lewis X (Le^X^) were immunohistochemically evaluated.

**Results:**

UC-associated carcinoma showed worse prognosis than sporadic colon carcinoma in all the cases, and exhibited a tendency to become more poorly differentiated when carcinoma invaded the submucosa or deeper layers than sporadic carcinoma. When the lesions were compared with sporadic carcinomas considering differentiation grade, reduced expression of CD44 extracellular domain in UC-associated carcinoma was apparent. Laminin-5γ2 and sialyl-Le^X ^expression showed a lower tendency in UC-associated carcinomas than in their sporadic counterparts. There were no differences in the numbers of tumor budding foci between the two lesion types, with no apparent relation to nuclear beta-catenin levels in IHC.

**Conclusions:**

UC-associated carcinoma showed poorer differentiation when the carcinoma invaded submucosa or deeper parts, which may influence the poorer prognosis. The invasive behavior of UC-associated carcinoma is more associated with CD44 cleavage than with basement membrane disruption or sialyl-Lewis-antigen alteration.

## Background

It is certain that patients with ulcerative colitis (UC) are more liable to develop a colon carcinoma than those without UC [1]. In our experience, UC-associated carcinoma tends to be more poorly differentiated when the carcinoma invades deeper than the submucosa, even if it is well differentiated in the lamina propria. Although the prognosis of UC-associated carcinoma had been regarded as grave in the past [2,3], clinicopathological studies suggested similarity to sporadic colorectal carcinoma [2-4]. However, it is still controversial, and the difference of invasion between UC-associated carcinoma and sporadic colon carcinoma has not yet been elucidated.

Focusing on factors for invasion, we earlier compared expression of cell adhesion molecules in UC-associated carcinomas with those in sporadic colonic adenocarcinomas, revealed significantly decreased expressions of CD44 and alpha-catenin and altered expression of beta-catenin in UC-associated lesions [5]. However, it was unclear whether or not UC-associated carcinoma has a more invasive behavior and whether or not carcinoma cell differentiation influences the biological behavior in UC-associated lesions. Therefore, in the present study, we tried to compare UC-associated carcinoma cases with sporadic colorectal adenocarcinoma counterparts, focusing on prognosis, tumor cell differentiation, tumor budding, and several protein expressions.

Recently, tumor budding has become considered a major malignant characteristic of colorectal carcinomas [6,7]. Defined as the presence of isolated single cells or small cell clusters (≤4 cells) scattered in the stroma at invasive fronts [6], this features loss of both glandular differentiation and cell cohesion that is crucial for the development of high-invasive properties. Tumor budding has been reported to be a risk factor for lymphatic involvement and lymph node metastasis in sporadic colorectal carcinomas [8,9]. However, the prognoses of patients with UC-associated carcinomas are not known.

Regarding cell adhesion molecules, the down-regulation of CD44 expression is reported to correlate with metastasis and poor prognosis in various types of carcinoma [10,11]. Recently, it was reported that CD44 reduction is caused in part by the proteolysis-based cleavage of its extracellular domain, which occurs in many malignant tumors [12]. Zo-1 and occludin are tight junction-associated proteins, which seal the cells together and prevent diffusion of solutes from the outside [13]. It was reported that both occludin and Zo-1 showed reduced expression in poorly differentiated gastrointestinal carcinoma [13].

When beta-catenin translocates into the nucleus and binds to the T-cell factor, it acts as a transcription factor for various genes [14], including matrix metalloproteinase-7 (MMP-7, matrilysin) [15], which is known to play roles in extracellular matrix degradation [16,17]. One of the main targets of MMP-7 in epithelial basement membranes is the laminin-5 isoform, formed by association of α3, β3, and γ2 chains. Specific cleavage of the laminin-5γ2 chain by members of the MMP family has been proposed to favor cell migration [17,18].

A modified blood group ABO/Lewis antigen, sialyl Lewis X (Le^X^) is present on the surfaces of human leukocytes [19,20]. It was also reported to be expressed in colonic carcinoma cells while sialyl 6-sulfo Le^X ^is characteristic of normal colonic epithelium [21], and it was considered to play an important role in metastasis through the binding to E-selectin on endothelial cells [22]. Increased expression of sialyl Le^X ^correlates with a poor prognosis in patients with colorectal carcinoma, demonstrating relations to the depth of tumor invasion, lymph node metastasis, lymphatic invasion, and the disease stage [23,24].

In the present study, expression of CD44 extracellular domain, Zo-1, occludin, MMP-7, and laminin-5γ2 in association with beta-catenin nuclear localization, and levels of sialyl Le^X ^were immunohistochemically investigated along with tumor budding in UC-associated and sporadic colon carcinomas, with the aim of elucidating differences in mechanisms of invasion and biological behavior between the two types of colon carcinomas in relation to prognoses.

## Methods

From the pathology files of Kitasato University East Hospital and three other affiliated hospitals, 15 cases of UC-associated colonic carcinoma were collected, and 12 of those 15 were located in the left hemicolon. Histologically, 6 cases were well differentiated adenocarcinoma, 4 cases being moderately differentiated adenocarcinoma, and 5 cases being poorly differentiated adenocarcinoma (including signet ring cell carcinoma). Duration of illness from onset to surgical resection was from 6 to 19 years (mean, 13.1 years). The lesions were histologically confirmed by three expert pathologists (TM, TY, and IO) according to the criteria of Riddell *et al*. [25].

### Prognosis analysis

A total of 300 consecutive cases of sporadic advanced adenocarcinoma were collected from the pathology files of the Kitasato University East Hospital from 1999 to 2000. The prognosis data of 15 UC-associated adenocarcinomas, and these 300 sporadic adenocarcinoma cases, were examined from their clinical charts. When calculating overall survival in the present study, only deaths from carcinoma were considered in the analysis.

### Histopathological examination

As sporadic counterparts, sporadic invasive adenocarcinoma cases of 5 times the number of UC-associated lesion cases were collected for the two histologic groups: 30 cases of well differentiated and 45 cases of moderately to poorly differentiated adenocarcinoma (moderately differentiated, 37 cases; poorly differentiated, 8 cases). The lesions were histologically confirmed by three pathologists (TM, TY, and IO), according to the World Health Organization histological typing [26]. In order to guarantee the material quality for immunohistochemical reaction and DNA analysis, the additional 75 cases from 2003 to 2004 were collected, not including the above 300 cases.

All histopathological materials were fixed in 10% buffered formalin and routinely processed for embedding in paraffin. From the invasive carcinoma lesions, both UC-associated and sporadic, paraffin blocks, including the deepest invasion parts, were chosen for examination. The differentiation of adenocarcinoma at both the surface and deep parts were histologically examined for each lesion.

Immunohistochemical staining of 4-micrometer-thick paraffin sections was performed using a commercial kit (EnVision+, Dako, Glostrup, Denmark). The primary antibodies used and methods for antigen retrieval are listed in Table 1. Microwave oven heating (500 W, 5 min, ×3 times) was done with a target retrieval solution (pH 9.0, Dako) for cytokeratin, and with a target retrieval solution (pH 6.0, Dako) for MMP-7, beta-catenin, laminin-5γ2, and sialyl Le^X ^to retrieve antigenic activity. Protease treatment (15 min, 37°C) was done for Zo-1 and occludin. The sections were subsequently incubated with the primary antibodies at 4°C overnight. After processing according to the manufacturer's protocols, 3,3'-diaminobenzidine was used as the final chromogen, and nuclei were counter-stained with hematoxylin or methyl green.

**Table 1:** Antibodies used for the immunohistochemical examination

Antibody	Clone	Source	Dilution	Antigen retrieval	Interpretation
CD44 extracellular domain (aminoterminal end)	Polyclonal, Ab64929	Abcam, Cambridge, MA	1/50	Not applied	Sinicrope's method*
Zo-1	Polyclonal, 61-7300	Zymed, South San Francisco, CA	1/100	Protease treatment (15 min, 37°C)	Linear or dot-like
Occludin	Polyclonal, 71-1500	Zymed	1/100	Protease treatment (15 min, 37°C)	Linear or dot-like
MMP-7	Monoclonal, 141-7B2	Daiichi Fine Chemical, Takaoka, Japan	1/200	Microwave treatment for 15 min (Dako target retrieval solution [pH 6.0])	Sinicrope's method*
Pancytokeratin	Monoclonal, MNF116	Dakocytomation, Glostrup, Denmark	1/50	Microwave treatment for 15 min (Dako target retrieval solution [pH 9.0])	For evaluation of tumor budding
beta-Catenin	Monoclonal, 14/beta-catenin	BD Transduction laboratories, Lexington, KY	1/200	Microwave treatment for 15 min (Dako target retrieval solution [pH 6.0])	Nuclear, more than 20% judged as positive; membranous and cytoplasmic, Sinicrope's method*
Laminin-5γ2	Monoclonal, 4G1	Dakocytomation	1/50	Microwave treatment for 15 min (Dako target retrieval solution [pH 6.0])	Sinicrope's method*
Sialyl Lewis X	Monoclonal, FH6	Ohtsuka Pharmaceutical, Tokushima, Japan	1/2000	Microwave treatment for 15 min (Dako target retrieval solution [pH 6.0])	Sinicrope's method*

• Sinicrope's method is described in Reference [27].

### Evaluation of tumor budding

Isolated single carcinoma cells and small cell clusters composed of fewer than five carcinoma cells were defined as tumor budding foci in the stroma at actively invasive tumor margins [6]. The numbers of tumor budding foci on pancytokeratin-stained slides were counted using a ×10 objective lens along the invasive front of carcinomas for at least 1.5 cm in length per case, after which the value per 1 cm was calculated.

### Expression of CD44 extracellular domain, MMP-7, laminin-5γ2, sialyl Le^X^, Zo-1, occludin, and beta-catenin

At the invasive fronts of carcinoma lesions, immunoreactivity for CD44 extracellular domain, MMP-7, laminin-5γ2, and sialyl Le^X ^was evaluated and semi-quantified according to the classification of Sinicrope *et al*. [27] focusing on both staining intensity and frequency of stained cells. The intensity was scored: weak, 1; moderate, 2; intense, 3. For frequency, positive cells were expressed as the percentage of the total number of cells in the invasive front and assigned to 1 of 5 categories: 0, <5%; 1, 6-25%; 2, 26-50%; 3, 51-75%; 4, >76%. Immunoreactivity scores for each lesion were calculated by multiplication of the values for the two parameters. For Zo-1 and occludin staining, the expression was observed at the cell surface of the lumen composed of adenocarcinoma cells. The expression pattern, linear or dot-like, was investigated. For beta-catenin, when more than 20% of the carcinoma cells showed nuclear immunoreactivity [28], the case was judged as nuclear beta-catenin positive (Table 1). Sinicrope's method, described above, was used for the beta-catenin membranous and cytoplasmic expressions [27].

### DNA extraction and sequencing analysis

DNA was extracted from formalin-fixed, paraffin-embedded tissue samples. Five 10-μm-thick sections were cut and deparaffinized in xylene before dehydration with 100% ethanol. After nuclear staining with hematoxylin, lesions were microdissected under a microscope with disposable scalpels, and DNA was extracted with an extraction kit (QIAamp DNA Mini Kit, Qiagen, Hilden, Germany). The collected samples were treated with proteinase K, and lysates were processed according to the manufacturer's protocols.

Examination of the exon 3 sequence of the beta-catenin gene was done according to the methods used by Saegusa *et al*. [29]. Briefly, amplification was achieved by polymerase chain reaction (PCR) using TaKaRa ExTaq polymerase (Takara Bio Inc., Ohtsu, Japan) in a volume of 20 μl with forward (5'-ATTTGATGGAGTTGGACATGG-3') and reverse (5'-TCTTCCTCAGGATTGCCTT-3') primers with the conditions: 33 cycles of 94°C for 0.5 min, 55°C for 1 min, and 72°C for 1 min. Subsequently, 5 μl aliquots of reaction products were applied to agarose gel electrophoresis to confirm the PCR reaction. As a negative control, water was supplied instead of template DNA for each examination.

The remaining 15 ml aliquots of PCR products were purified using a QIAquick PCR Purification Kit (Qiagen) and sequenced using a BigDye Terminator Cycle Sequencing Kit (Applied Biosystems, Foster City, CA) according to the manufacturers' protocols. The DNA sequence data were collected and analyzed on an ABI Prism 3100 automated DNA sequencer (Applied Biosystems). If a mutation was found using the forward PCR primer, the reverse PCR primer was used to confirm the mutation.

### Statistical analysis

Statistical analysis was performed using the χ^2 ^and Mann-Whitney *U *tests. When more than 3 groups were compared, the Kruskal Wallis test with the Mann-Whitney *U *test was applied. Prognosis was analyzed by the Kaplan-Meier method with the log-rank test. All calculations were performed using SPSS computer software (SPSS Inc., Chicago, IL, USA), and *P*-values less than 0.05 were considered statistically significant.

## Results

**Figure 1:**
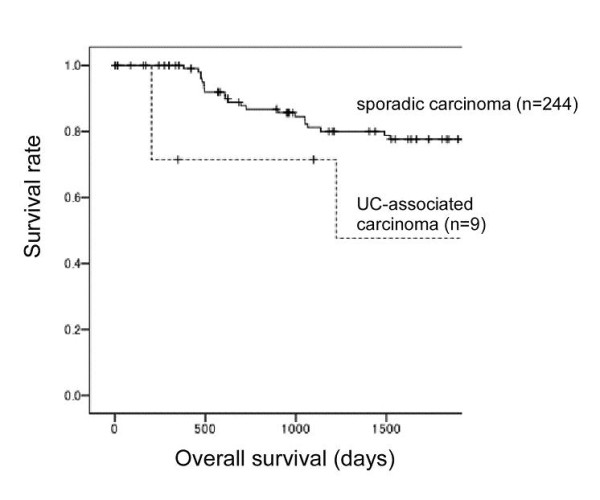
**Prognoses were analyzed for cases of ulcerative colitis (UC)associated and sporadic colon carcinomas of stages 2 and 3**. For all cases, UC-associated carcinoma showed worse overall survival than sporadic carcinoma (*P *= 0.010). When cases were limited to well differentiated carcinomas, no significant difference was shown (*P *= 0.136). Similarly, the cases limited to moderately to poorly differentiated ones, no significant difference was shown (*P *= 0.071).

Prognosis was analyzed for Stage 2 and 3 cases (9 UC-associated and 244 sporadic carcinomas). As for overall survival of all cases, UC-associated carcinoma showed worse prognosis than sporadic carcinoma (*P *= 0.010) (Figure 1). However, when the cases were limited to well differentiated carcinomas, no significant difference was shown (*P *= 0.136). Similarly, the cases limited to moderately to poorly differentiated ones, no significant difference was shown (*P *= 0.071). Clinicopathological data for the 15 UC-associated and 75 sporadic colon carcinoma cases are summarized in Table 2. There were no differences in the rates of lymph node metastasis between the 2 groups (Table 2). In comparison between the surface and deep parts of the lesions, UC-associated carcinomas tended to become more poorly differentiated when the carcinoma invaded the submucosa or deeper (Table 3). The rate of the cases which showed poorer differentiation at the deep part was significantly higher in UC-associated carcinoma (10 of 15 cases, 66.7%) than in sporadic colon carcinoma (25 of 75 cases, 33.3%) (*P *= 0.033).

**Table 2:** Clinicopathological summary of the cases

	UC-associated	Sporadic	*P *value
Site			
Left side	12	41	0.068
Right side	3	34	
Histological type			
Well differentiated	6	30	
Moderately differentiated	4	37	
Poorly differentiated (including signet-ring cell carcinoma)	5	8	
Depth of invasion			
Up to MP	2	12	0.79
SS/A or deeper	13	63	
Lymph node metastasis			
Negative	5	31	0.56
Positive	10	44	
Stage			
I	2	9	0.14
II	3	22	
III	9	44	
IV	1	0	

UC-ulcerative colitis; MP-muscularis propria; SS-subserosa; A-adventitia.

**Table 3:** Comparison of histologic type between mucosa and deeper parts (submucosa or deeper) of cancer lesion

		UC-associated (*n *= 15)	Sporadic (*n *= 75)	*P *value
Mucosa area	Deeper area			
Well	Well	2	25	
Well	Moderately	5	18	
Well	Poorly	2	0	
Moderately	Moderately	1	20	
Moderately	Poorly	3	7	
Poorly	Poorly	2	5	0.0062*

Poorer differentiation in deep parts**		10 (66.7%)	25 (33.3%)	0.033*

UC - ulcerative colitis; *n *- number of cases.

* χ^2 ^test,

** Cases which were shown to be well differentiated at the mucosa and moderately to poorly differentiated at the deep areas, and showed moderately differentiated at mucosa and poorly differentiated at the deep areas.

**Figure 2:**
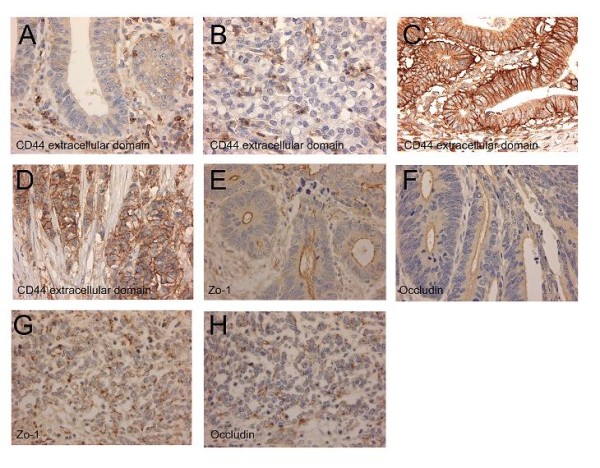
**Representative photographs of CD44 extracellular domain, Zo-1, and occludin expression**. CD44 extracellular domain is not expressed at the cell membrane of the invasive part of ulcerative colitis (UC)-associated well differentiated carcinoma (A) and a poorly differentiated carcinoma (B). On the other hand, in a case of sporadic well differentiated (C) and a case of sporadic poorly differentiated adenocarcinoma (D), membranous expression of CD44 extracellular domain is observed. Linear expression of Zo-1 (E) and occludin (F) is seen at the luminal surface in a case of UC-associated well differentiated carcinoma. However, in a UC-associated poorly differentiated case, dot-like expression of both Zo-1 (G) and occludin (H) is observed on the cell surface.

**Figure 3:**
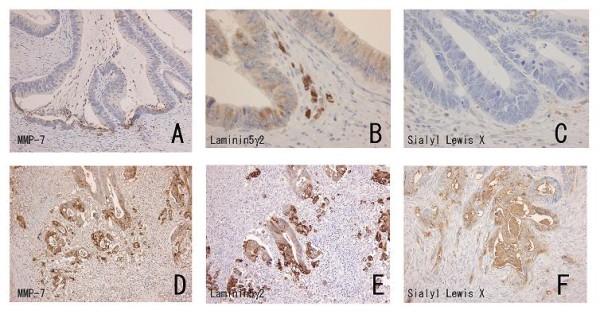
**Representative expression of matrix metalloproteinase-7 (MMP-7), laminin-5γ2, and sialyl Lewis X (Le^X^) in an ulcerative colitis (UC)-associated colon carcinoma case (A-C) and a sporadic colon carcinoma case (D-F)**. While MMP-7 expression is low in the UC-associated carcinoma with limited tumor budding (A), strong expression is seen in a sporadic carcinoma with frequent tumor budding (D). Laminin-5γ2 expression observed in invasive fronts of both UC-associated (B) and sporadic (E) cases, especially at tumor budding foci. Note that while no expression of Le^X ^is apparent in a UC-associated case (C), strong immunoreactivity is evident in the sporadic lesion (F).

The lesions were divided according to their differentiation, and UC-associated and sporadic carcinomas were compared in each differentiation category. CD44 extracellular domain showed lower expression in UC-associated carcinoma than in sporadic carcinoma, in both the well-differentiated category and the poorly differentiated category (*P *= 0.010, *P *< 0.001, respectively) (Figure 2) in invasive fronts. As the colon carcinoma cases would generally be divided into two categories: well to moderately differentiated and poorly differentiated cases [26], comparison between the 2 categories revealed that CD44 extracellular domain expression was significantly higher in sporadic well to moderately differentiated carcinoma than in the UC-associated counterparts (*P *= 0.002). There were no differences in tumor budding and MMP-7 expression. Laminin-5γ2 and sialyl Le^X ^both showed higher expression in sporadic carcinomas than in their UC-associated counterparts, without statistical significance (Table 4) (Figure 3). In division into the 2 categories, sialyl-Le^X ^expression was significantly higher in sporadic well to moderately differentiated carcinoma than in their UC-associated counterparts (*P *= 0.016).

**Table 4:** Tumor differentiation and budding, and expression of CD44 extracellular domain, MMP-7, laminin-5γ2, and sialyl Le^X ^in invasive fronts

	*n*	Tumor budding (/cm)	CD44	MMP-7	Laminin-5γ2	Sialyl Le^X^
Sporadic cancer						
Well differentiated	30	206.7 ± 293.9	5.5 ± 2.8*	4.2 ± 3.4	5.2 ± 2.7	3.2 ± 3.3
Moderetely differentiated	37	264.0 ± 240.6	5.1 ± 2.6	4.4 ± 3.4	5.7 ± 2.7	4.2 ± 3.1
Poorly differentiated	8	395.8 ± 280.5	6.6 ± 2.6**	2.6 ± 2.9	4.6 ± 3.5	1.1 ± 0.8
UC-cancer						
Well differentiated	6	146.2 ± 239.4	2.2 ± 1.5*	3.8 ± 2.8	4.0 ± 2.6	1.3 ± 2.3
Moderately differentiated	4	157.0 ± 178.7	2.8 ± 1.5	4.7 ± 1.5	5.0 ± 1.2	1.0 ± 0.0
Poorly differentiated	5	145.8 ± 213.4	1.2 ± 1.6**	1.0 ± 1.0	1.8 ± 2.0	1.4 ± 1.5

MMP - matrix metalloproteinase; *n *- number of cases; Le^X ^- Lewis X; UC - ulcerative colitis.

* P = 0.010

** *P *< 0.001

Expression of both Zo-1 and occludin was observed as lines or dots on the apical cell surface of a lumen or cell adhesion site. At the invasive fronts of UC-associated carcinoma of the 6 cases of well differentiated carcinoma, Zo-1 and occludin were expressed as a line pattern in 5 (83.3%) and a dot pattern in 1 (16.7%), respectively; and of the 9 cases of moderately to poorly differentiated carcinoma were expressed as a line pattern in 5 (55.6%) and a dot pattern in 4 (44.4%). On the other hand, all 30 (100%) well differentiated sporadic lesions showed linear expression of Zo-1 and occludin. For the 45 moderately to poorly differentiated cases, 36 (80.0%) showed a linear expression and 9 (20.0%) showed a dot pattern expression (Figure 2). There were no significant differences between UC-associated and sporadic carcinomas.

**Figure 4:**
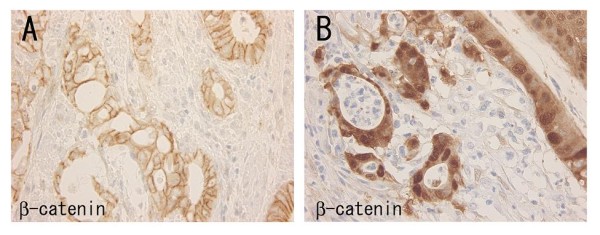
**Expression of beta-catenin in a case of ulcerative colitis-associated carcinoma (A) and a case of sporadic colon carcinoma (B)**. Strong membranous expression is observed in the UC-associated carcinoma (A). Nuclear and cytoplasmic expression is seen in the sporadic colon carcinoma, instead of membranous expression (B).

There were no differences in numbers of tumor budding foci among the sporadic carcinomas of the right and left hemicolon and the UC-associated carcinomas (Table 5). In addition, there were no differences in tumor budding between nuclear beta-catenin negative and positive cases of sporadic carcinoma. Cytoplasmic beta-catenin expression was significantly stronger in nuclear positive than in nuclear negative cases of sporadic carcinoma (Table 6) (Figure 4). In the UC-associated carcinomas, 1 of 2 nuclear beta-catenin positive lesions was a signet-ring cell carcinoma mixed with mucinous carcinomas. Therefore, comparison between nuclear beta-catenin negative and positive lesions in UC-associated carcinoma was not considered appropriate.

**Table 5:** Tumor budding in UC-associated and sporadic colon carcinomas

	*n*	Foci of tumor budding (/cm)
Sporadic cancer, right	34	278.4 ± 316.1
Sporadic cancer, left	41	235.9 ± 225.9
UC cancer	15	148.9 ± 200.9

UC - ulcerative colitis; *n - *number of cases.

**Table 6:** Relation among beta-catenin expression and tumor budding

	*n*	Foci of tumor budding (/cm)	β-catenin expression membranous	β-catenin expression cytoplasmic
Sporadic cancer				
β-catenin nuclear expression negative	26	248.5 ± 255.6	5.8 ± 2.8	4.2 ± 2.0*
β-catenin nuclear expression positive	49	258.7 ± 279.0	4.9 ± 2.9	6.6 ± 2.3*
UC cancer				
β-catenin nuclear expression negative	13	170.1 ± 208.4	5.2 ± 3.8	3.2 ± 1.9
β-catenin nuclear expression positive	2	11.5 ± 16.3	3.0 ± 0.0	6.0 ± 2.8

UC - ulcerative colitis; *n - *number of cases.

* *P *< 0.001

Mutations in beta-catenin exon 3 were found in 1 of 32 (3.1%) sporadic carcinomas of the right and 1 of 41 (2.4%) of the left hemicolon, but none were found in the 7 cases of UC-associated carcinomas examined. PCR or sequencing reactions failed in 11 cases, probably due to the poor quality of the extracted DNA.

## Discussion

In the present study, for overall cases, the prognosis of UC-associated carcinoma was worse than that of sporadic colon carcinoma. However, significant difference was not shown when the cases were limited to the same tumor differentiation. Because the UC-associated carcinoma cases contained much poorly differentiated cases, and since the number of cases was small, it was difficult to conclude that the prognosis of UC-associated carcinoma is definitely worse than that of its sporadic counterpart. However, the character that UC-associated carcinoma showed poorer differentiation at the invasion area in the submucosa or deeper parts may influence the prognosis.

The frequency of beta-catenin gene mutation was low in both UC-associated and sporadic carcinomas. In the literature, the frequency in sporadic colorectal carcinoma has varied from the 0% reported by Garinis *et al*. [30] to the percentages of 22% and 26%, respectively, described by Mirabelli-Primdahl *et al*. [31] and Aust *et al*. [32]. In contrast, Aust *et al*. [32] found no beta-catenin mutations in UC-associated carcinomas. Garinis *et al*. [30] examined mutations with the PCR-single strand conformation polymorphism method while Mirabelli-Primdahl *et al*. [31] and Aust *et al*. [32] used PCR-direct sequencing. Although the frequency of beta-catenin mutations thus appears to vary, in the present study, nuclear expression did not show any positive relation with tumor budding, a morphological feature of invading carcinomas. We conclude that the role of beta-catenin in invasion may be limited.

In our previous study, it was shown that expression of the CD44 standard form, variant 3, and variant 6 was reduced in UC-associated neoplasia compared to their sporadic counterparts [5]. In the present study, CD44 extracellular domain expression was significantly lower in UC-associated carcinoma than in its sporadic counterparts. Not the splicing variant but CD44 extracellular domain cleavage is considered to play a critical role in CD44-madiated tumor cell migration by regulating the interaction between CD44 and extracellular matrix [33,34]. It may play a key role in invasion of UC-associated carcinoma and its loss may be related with poor prognosis. On the other hand, tight junction associated proteins showed no difference. Although lesions with poorer differentiation showed reduced expression (dot pattern) of Zo-1 and occludin, the barrier function of tight junction may be essential for epithelial cells including carcinoma to maintain the microenvironment.

The expression of both laminin-5γ2, a marker of basement membrane disruption, and sialyl Le^X^, a cell surface antigen suggestive of a poor prognosis, showed here paradoxically lower tendency in UC-associated carcinomas than in sporadic counterparts. In invasion of UC-associated carcinoma, adhesion between CD44 molecule and extracellular matrix might be more important than basement membrane disruption or the alteration of sialyl Lewis antigens.

The average figures of tumor budding tended to be lower in UC-associated carcinomas than those in sporadic carcinomas, even in moderately to poorly differentiated groups. This may not be concordant with the findings that UC-associated carcinoma showed poorer differentiation in the submucosa or deeper parts. However, histologically, different from sporadic carcinomas, poorly differentiated UC-associated carcinoma cells often tended to show trabecular arrangement or much mucous production, not showing so-called tumor budding. Therefore, this discordance may be related with characteristics of invasion of UC-associated carcinoma.

Because UC-associated carcinoma showed poorer differentiation when it invaded the submucosa or deeper layers, early detection of UC-associated neoplastic lesions before submucosal invasion is considered important. Recent advances in dye spraying and magnifying endoscopy for the colon have made it possible to detect early neoplastic lesions in UC follow-up patients [35,36]. Such endoscopic detection should allow great improvement in outcome for affected patients.

## Conclusions

UC-associated carcinoma showed poorer differentiation when the carcinoma invaded submucosa or deeper, which might influence its poorer prognosis. Its invasive behavior is more associated with CD44 cleavage than with basement membrane disruption or sialyl Lewis antigen alteration.

## Competing interests

The authors declare that they have no competing interests.

## Authors' contributions

TM carried out the experiments, collected and interpreted the data, and wrote the manuscript. YN carried out the molecular experiments, and KA performed immunohistochemical staining and evaluation. TY, MK, and NN analyzed the data. IO contributed conception and designed the study. All authors read and approved the final manuscript.
